# Behaviour of Telomere and Telomerase during Aging and Regeneration in Zebrafish

**DOI:** 10.1371/journal.pone.0016955

**Published:** 2011-02-09

**Authors:** Monique Anchelin, Laura Murcia, Francisca Alcaraz-Pérez, Esther M. García-Navarro, María L. Cayuela

**Affiliations:** Telomerase, Aging and Cancer Group, Research Unit, Department of Surgery, CIBERehd, University Hospital “Virgen de la Arrixaca”, Murcia, Spain; National Institute on Aging, United States of America

## Abstract

Telomere length and telomerase activity are important factors in the pathobiology of human diseases. Age-related diseases and premature aging syndromes are characterized by short telomeres, which can compromise cell viability, whereas tumour cells can prevent telomere loss by aberrantly upregulating telomerase. The zebrafish (*Danio rerio*) offers multiple experimental manipulation advantages over other vertebrate models and, therefore, it has been recently considered as a potential model for aging, cancer, and regeneration studies. However, it has only partially been exploited to shed light on these fundamental biological processes. The aim of this study was, therefore, to investigate telomere length and telomerase expression and activity in different strains of zebrafish obtained from different stock centres to determine whether they undergo any changes during aging and regeneration. We found that although both telomerase expression and telomere length increased from embryo to adulthood stages, they drastically declined in aged fish despite telomerase activity was detected in different tissues of old fish. In addition, we observed a weaker upregulation of telomerase expression in regenerating fins of old fish, which well correlates with their impaired regeneration capacity. Strikingly, telomeres were elongated or maintained during the fin regeneration process at all ages and after repeated amputations, likely to support high cell proliferation rates. We conclude that the expression of telomerase and telomere length are closely related during the entire life cycle of the fish and that these two parameters can be used as biomarkers of aging in zebrafish. Our results also reveal a direct relationship between the expression of telomerase, telomere length and the efficiency of tissue regeneration.

## Introduction

During the last decade, the research on the processes of human aging and tumour formation has grown considerably in order to prevent or halt the progression of aging and cure age-associated diseases and cancer. Compared with other models, the zebrafish offers multiple experimental manipulation advantages and has recently been considered as a potential model for aging, cancer, and regeneration study [1]–[5].

It is well established that the accumulation of cellular damage is at the origin of both cancer and aging. One of the best known molecular mechanisms of aging is the progressive attrition of telomeres with age both in human and mice [6], [7]. Recent studies in the mouse model showed that telomerase might have a fundamental role in tumour growth and survival [8]. Furthermore, short telomeres and defective telomerase activity have been involved in the pathobiology of several age-related diseases and premature aging syndromes, such as dyskeratosis congenita and aplastic anaemia [9].

Telomeres are nucleoprotein complexes at the end of the chromosomes [10] which consist of TTAGGG repeats and several associated proteins [11]. As conventional DNA-dependent DNA polymerase fails to fully replicate the ends of linear molecules, 50–200 bp of telomeric DNA repeats are lost with every round of cell division. At the cell level, cell cycle arrest, cell senescence or crisis occur when telomeres reach a critical size, compromising cell viability. The telomerase, a reverse transcriptase compensates for the telomere loss in those cell types where it is expressed [12]–[15], but its level of activity in most adult tissues is not sufficient to counteract telomere shortening with aging [16]. In human, telomerase expression is principally restricted to highly proliferating cells (germ cells and progenitor/stem cells) in adults, whereas telomerase activity is expressed during human embryonic development [17]–[19] and it is highly detectable in immortalized cells and many tumour cell tissues [20]–[22].

Although human organs have a limited ability to heal and regenerate damaged or lost tissue, the zebrafish retains remarkable regenerative abilities in retina, fins, heart, spinal cord and other tissues to later advanced ages [23]–[27]. Moreover, the zebrafish has constitutively abundant telomerase activity in somatic tissues from embryos to aged adults [5], [28]. Notably, a recent study on various tissues from aquatic species including the zebrafish suggests that telomerase may be important for tissue renewal and regeneration after injury rather than for overall organism longevity [29]. Despite this substantial advance, the advantages of this vertebrate model to decipher the role of telomerase in regeneration, aging and cancer have only been partially exploited. Therefore, the aim of this study was to investigate telomere length and telomerase expression and activity from different strains of *Danio rerio* obtained from different stock centres and to determine whether it undergoes any changes during aging. We found that the expression of telomerase and the telomere length are closely related during the entire life cycle of the fish and that these two parameters can be used as biomarkers of aging in zebrafish. Our results also reveal a direct relationship between the expression of telomerase, telomere length and the efficiency of tissue regeneration.

## Results

### Telomerase Expression Increase during Development and Decrease with Aging

**Figure 1:**
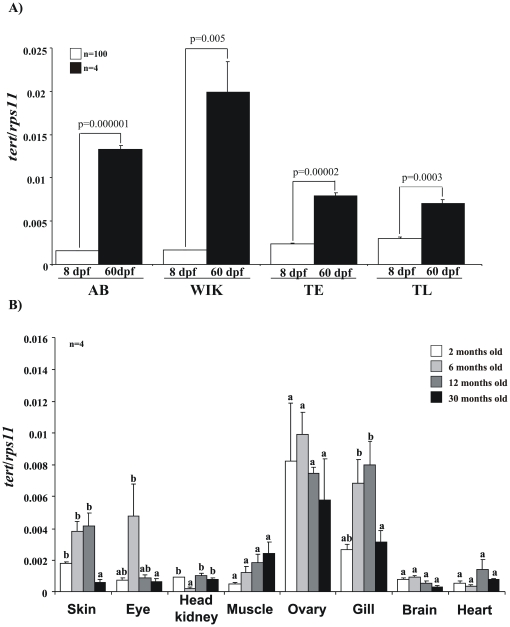
Dynamic of TERT gene expression in the zebrafish. The mRNA levels of *tert* gene were determined by real-time RT-PCR in larval and juvenile stages of the indicated genotypes (**A**) and in different tissues of 2–30 month-old fish of the AB genotype. Gene expression is normalized against *rps11*. Each bar represents the mean ± S.E. from 100 pooled animals for larvae and 4 individual fish for all the rest (**A,B**) and triplicate samples.

We first analyzed the expression of TERT in four different zebrafish genotypes at larval and juvenile stages and also in several organs throughout life (Fig. 1). The results showed a significant increase of telomerase expression between the larval and the juvenile stage of zebrafish (Fig. 1A) in the four analyzed genetic backgrounds. In addition, telomerase expression also increased during the developmental stages in all organs, but drastically declined in older fish with the exception of the muscle (Fig. 1B).

### Telomerase Activity is detected in Zebrafish of all Ages

Because telomerase activity was regulated at different levels, we examined the correlation between zebrafish TERT transcript levels and telomerase activity, assayed by conventional **T**elomerase **R**epeat **A**mplification **P**rotocol (TRAP) and Q-TRAP (**Q**uantitative), which has been reported to be a rapid and accurate assay for the quantification of telomerase activity [30], [31].

**Figure 2:**
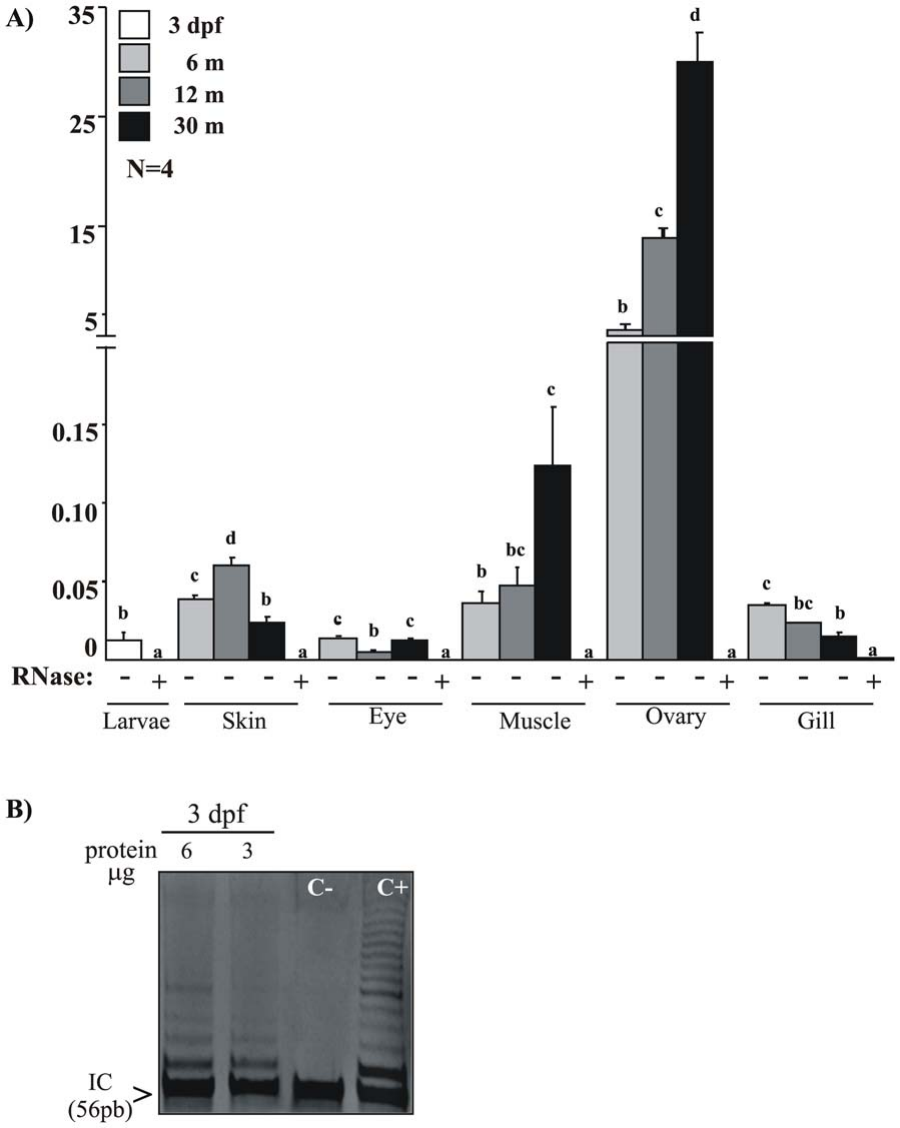
Very old fish have telomerase activity. Telomerase activity was measured quantitatively and qualitatively in whole zebrafish embryos (3 days post-fertilization, dpf, n = 100) and in several organs from adults with different ages (6, 12 and 30 months old, n = 4). **A,** Q-TRAP assay using 1 µg of protein extract. Results are expressed as the mean value ± S.E. from triplicate samples relative to telomerase-positive cells. Different letters denote statistically significant differences between different ages of each sample according to a Tukey test. **B,** TRAP assay using protein extract from whole zebrafish embryos. A ladder of bands indicates the presence of telomerase activity. The lowest band (56 pb) is the internal control (IC). Lanes C− and C+ correspond to telomerase- negative and positive controls, respectively. In all cases, to confirm the specificity of the assay, a negative control is included for each sample, treated with 1 µg of RNAse at 37°C for 20 min. The Q-TRAP assay was also performed using 0.1 µg of protein extract and the same relative results were obtained (data not shown).

Telomerase activity was detected in larvae as well as in different tissues from different age fish, including 30-month-old fish (Fig. 2). To confirm the specificity of the TRAP assay in zebrafish samples, we pretreated the protein extracts with RNase. This treatment abolished telomerase activity and served as a negative control. We obtained a good correlation between telomerase expression and telomerase activity in most samples (Figs. 1B and 2A). Notably, we found very high telomerase expression and activity in ovary, even though very old female (30 months old) are not fertile. The muscle showed increased telomerase activity during aging. However, we found a significant decrease in telomerase activity during aging (between 12 and 30 months) in gill and skin, and an invariable activity in the eye.

### The Transcription Factors c-Myc and NF-κB Activate the Zebrafish Telomerase Promoter

Telomerase expression is modulated by both genetic and epigenetic mechanisms [reviewed by 32,33] even in zebrafish [34]. The primary mechanism of telomerase regulation however, appears to be the transcriptional control of TERT via a complex network of transcription factors.

**Figure 3:**
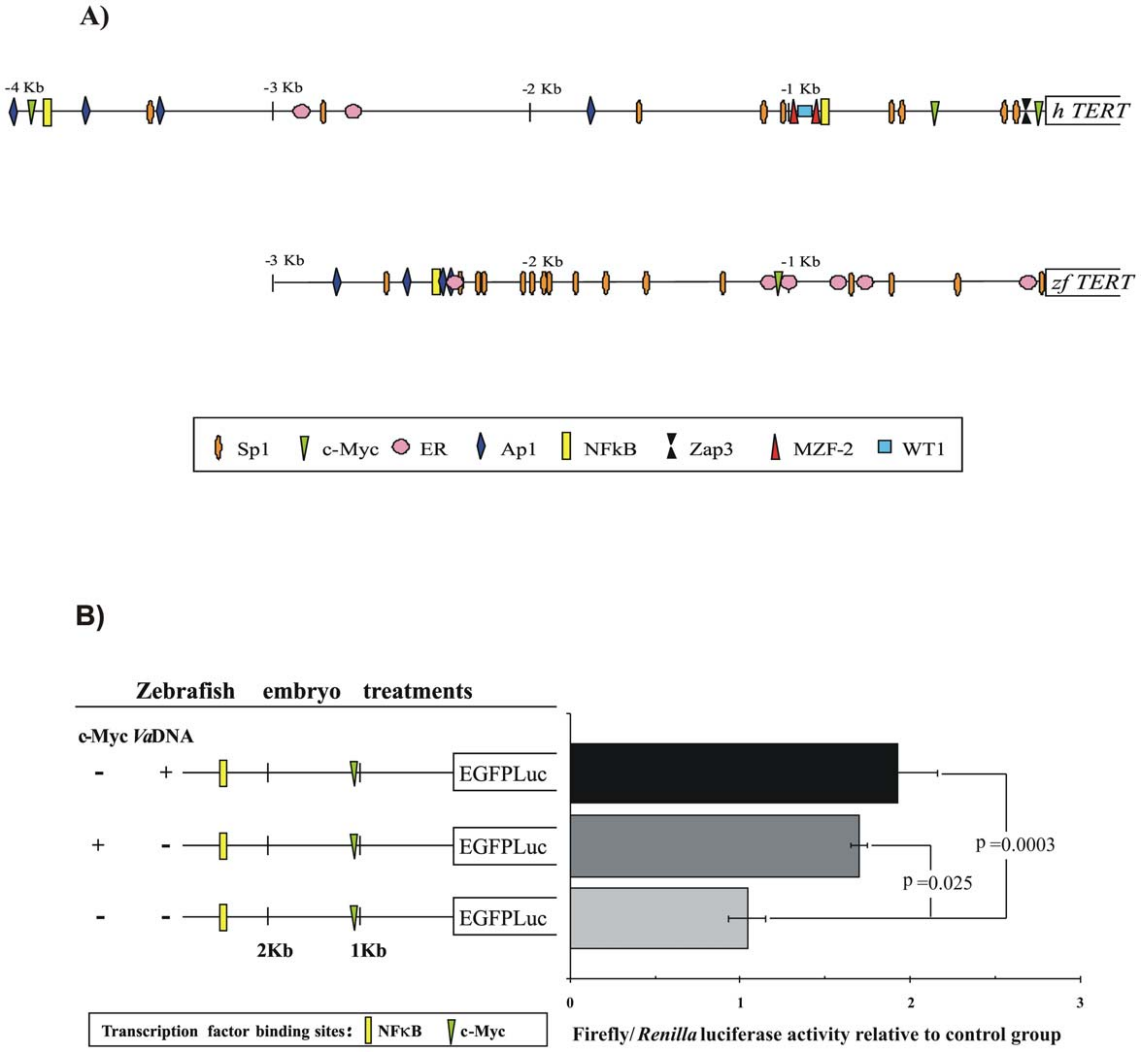
A, Putative regulatory elements of human and zebrafish *TERT* promoter. The putative binding sites of different transcription factors in the zebrafish telomerase promoter were determined by using the TESS (Transcription Element Search System): http://www.cbil.upenn.edu/cgi-bin/tess/tess. The information about the putative transcription factor binding site in the human telomerase promoter was previously described by *Pericuesta et al. 2006*. **B, The transcription factors c-Myc and NFkB regulate the expression of the zebrafish** ***TERT*** **gene.** The *zfTERT* promoter-EGFPLuc reporter constructs are shown on the left, while the relative luciferase activity of each construct is shown on the right. Each bar represents the mean ± S.E. from triplicate samples. Different letters denote statistically significant differences between the group according to a Tukey test. A promoterless construct and a *Renilla* luciferase expression vector were used in all cases as blank and internal controls, respectively.

To investigate the relationship between zebrafish and human *TERT* promoter regulation, a homology comparison was carried out between 3996 bp of the *hTERT* (AF097365) and the 2856 pb of the *zfTERT* (ENSDARG00000042637) sequences upstream of the coding sequence (Fig. 3). We have analyzed the presence of the transcription factor binding sites (Sp1, c-Myc, NF-*κ*B, ER, Ap1, Zap3, WT1 and MZF2). The order and localization of these sites relative to the position of the initiation codon is however not conserved between the *hTERT* and *zfTERT* promoter (Fig. 3A). The zebrafish TERT sequence had multiple putative Sp1 binding sites between −1 and −3 Kb, whereas in human the promoter has at least nine Sp1 binding sites along the 4 Kb promoter. On the other hand, the *zfTERT* promoter has only one putative c-Myc binding site located in the −1020 bp position, meanwhile the human promoter had 3 c-Myc located along the promoter. Another difference is that the zebrafish promoter had more putative ER binding sites than the human one, while it only had one NF-*κ*B located in the −2321 pb position versus two in the human promoter. Nevertheless, the most significant difference between both promoters is the absence of the Zap3, MZF-2 and WT1 inhibitors in the zebrafish promoter.

The above results prompted us to investigate the impact of c-Myc and NF-κB in the expression of zebrafish telomerase. c-Myc is reported to be a dominant transcriptional activator for hTERT expression [35]–[39] and it is also known that c-Myc activates the *hTERT* promoter twice in the HeLa cell line [40]. It is also known that NF-κB transactivates c-Myc to stimulate hTERT promoter activity [reviewed by 41]. To determine whether these transcription factors are involved in the activation of the *zfTERT* promoter, we performed a luciferase assay using zebrafish embryos. One- to eigth-cell zebrafish embryos were microinjected with zfpTERT-EGFPLuc, alone or in combination with c-Myc or with genomic bacterial DNA (*Va*DNA) to induce the activation of NF-κB [42]. We observed that both c-Myc and NF-κB were able to activate the zfTERT promoter (Fig. 3B), as has been shown for its human counterpart [41].

### Telomere Length and Telomere Shortening in Zebrafish

In normal somatic cells, telomeres shorten with each cell division. Telomerase activity is thought to be required for telomere length maintenance in mammals, as it is in yeast. Telomerase deficient mice show telomere shortening [43]. At present, the actual length of zebrafish telomeres and how telomere length changes with the age of this species are both unknown.

**Figure 4:**
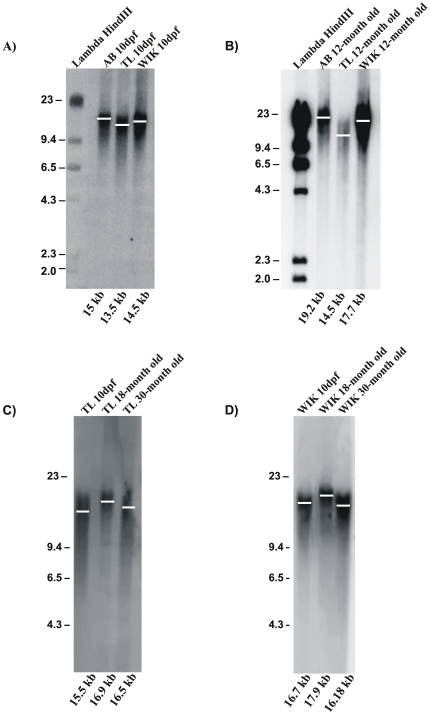
Dynamic of telomere length in zebrafish assayed by TRF. A representative TRF shows telomere length of different zebrafish background from larvae (**A**) and adults (**B**), and in TL (**C**) and WIK (**D**) zebrafish genotypes throughout their life cycles. Telomere length (Kb) is calculated using a quantitative algorithm involving the signal intensity of each telomere smear and the migration of the lambda HindIII ladder (lane 1). 3–5 independent TRF experiments were perform. We used a zebrafish embryos (10 dpf, n = 100) and adult fish for each strain with different ages (12, 18 and 30 months old, n = 3–4).

To determine telomere zebrafish length and if telomere shortening occurs even though telomerase activity is present in somatic tissues throughout their lives, we used both the TRF-Southern blot and the Q-FISH techniques. TRF analysis revealed that telomere length in adult zebrafish is around 16 kb±3.5 Kb (Fig. 4), as reported by others [5], [28], [29], [44]. TL zebrafish background showed shorter telomere that the other background at all studied ages (Fig. 4). However, we also found that telomere length increased during development until a critical time (around 18 months of age) when it started to decrease (Fig. 4C–D).

**Figure 5:**
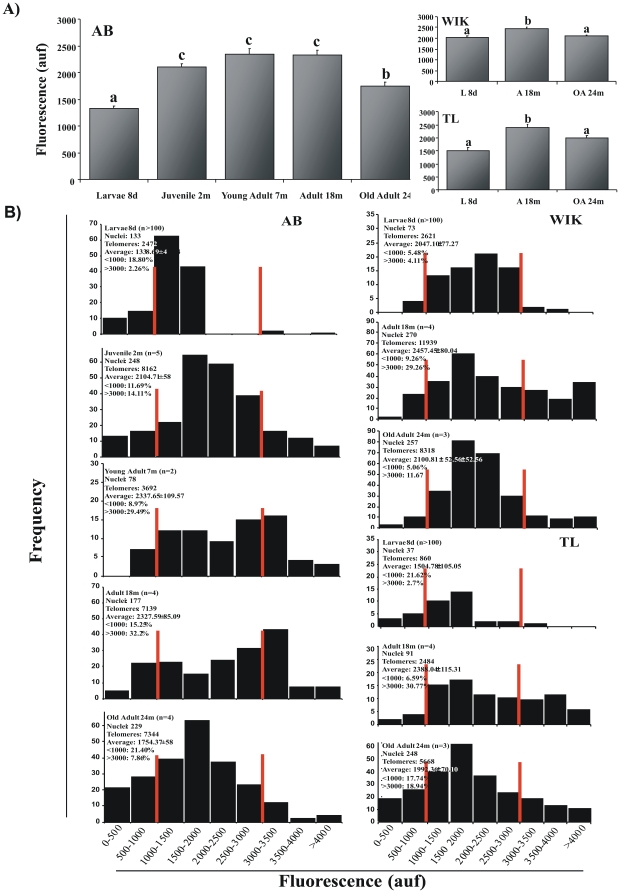
Dynamic of telomere length assayed by Q-FISH. **A**, Graph showing the mean telomeric fluorescence values. Data are mean values ± S.E. and statistical significance was assessed using the Tukey test (*p<0.05*). **B**, Histograms showing telomere fluorescence frequencies. The red lines demarcate both the shortest (<1000 auf) and the longest telomere percentage (>3000 auf) clearly illustrating telomere lengthening throughout the life cycle to adulthood and shortening in old age.

To further confirm the results obtained using the TRF assay, we analyzed telomere length variation throughout the life of the zebrafish using the more sensitive and specific Q-FISH method. The low detection limit of Q-FISH (<0.1 kb of telomere repeats) allows quantification of very short telomeres (1–3), and the use of a fluorescent peptide nucleic acid (PNA) probe against telomeric repeats facilitates the specific labelling of telomeres at the single cell level [45]–[47]. In mammals, a recent study showed that mean telomere length values obtained using the conventional Q-FISH analysis correlated excellently with telomere length values obtained using Q-FISH in interphase nuclei [48]. We used Q-FISH in interphase nuclei to determine the mean telomere length values of the whole organism in three zebrafish genotypes (AB, WIK and TL) at different ages. These results allowed us to generate telomere length frequency histograms and determinate the percentage of cells with very short telomeres (<1000 a.u.f.) and very long telomeres (>3000 a.u.f.). Our results were consistent with those obtained previously by TRF analysis (Fig. 4). In the case of the AB genotype, the telomere length showed a statistically significant increase between the larvae stage at 8 days post fertilization (1338.70 a.u.f. ±45.14) and the juvenile stage at 2 months of life (2104.75 a.u.f. ±58.08). After that, the telomere length of the young adult zebrafish at 7 months of life showed a higher mean value (2337.65 a.u.f. ±109.57), which remained unchanged at the adult age of 18 months of life (2327.59 a.u.f. ±85.09). Finally, we observed a very significant decrease (1754.37 a.u.f. ±58.47) in 24 month-old fish. (Fig. 5A) Telomeres lengthening during development as well as its shortening in old fish were very well illustrated by the telomere length frequency histograms (Fig. 5B). In the three genotypes, the percentage of very long telomeres (>3000 a.u.f.) showed its highest value at the adult age (18 months old) and fell drastically at old age (24 months old). Thus, we found 32.20% versus 7.86% in AB, 29.26% versus 11.67% in WIK, and 30.77% versus 18.90% in TL. As shown in Fig. 5A and 5B, both average telomere length and telomere length frequency histograms were very similar in the different zebrafish genotypes. However, the TL background always showed an average telomere length shorter than the other strains. In general, zebrafish has a telomere length in the range of 12.13 kb±0.41 to 22.28 kb±0.04.

### Impaired Caudal Fin Regeneration with Aging

**Figure 6:**
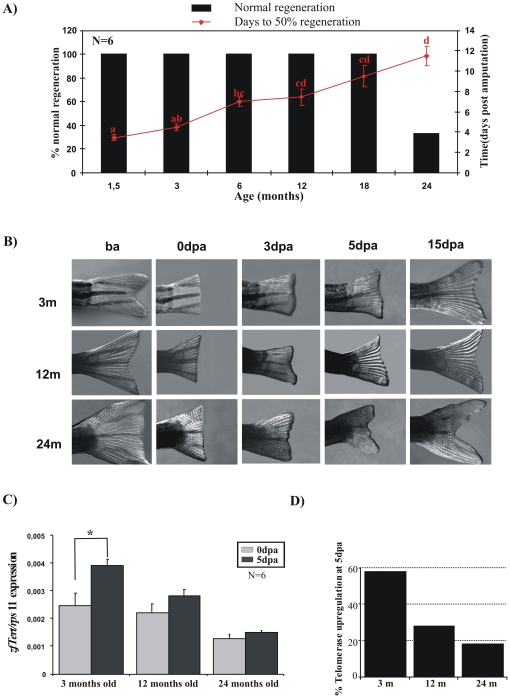
Old fish show impaired regeneration response. **A**, Percent fin regeneration was determined based on the area regrowth divided by the original fin area, n = 6 (ba: before amputation, dpa: days post-amputation). Black bars show normal caudal fin regeneration and red line indicate the days to reach 50% of the regeneration. **B**, Representative image of the fin regeneration assay. **C**, Analysis of *TERT* expression in regenerating fin 0 and 5 dpa, assayed by real time RT-PCR. Gene expression is normalized against *rps11*. Data are mean values ± S.E. D. Percentage of telomerase upregulation at 5 dpa.

The zebrafish, as most aquatic animals, has a high capacity for tissue regeneration and the importance of telomerase upregulation during the repair of injured tissues has already been tested in other fish species [29]. To establish this relationship in zebrafish, we performed a caudal fin regeneration assay to assess the regeneration process at the different zebrafish ages. Our results (Fig. 6A) showed that old adult zebrafish have a lower growth rate, reaching 50% of their caudal fin regeneration 12 days post-amputation (dpa) and did not display normal caudal fin regeneration (Fig. 6B). These results suggest a direct correlation between the lower expression of telomerase and the impaired caudal fin regeneration in older fish. We analyzed the telomerase expression from caudal fin tissues of 3, 12 and 24 month-old zebrafish before and after amputation, by real-time RT-PCR. We observed a significant decrease of telomerase expression with aging (Fig. 6C), confirming our previous results. However, amputation resulted in a significant telomerase upregulation in 3 month old zebrafish (58% of upregulation compared with basal expression) and caused weak increased telomerase expression in 24-month old fish (18% of upregulation) at 5dpa (Fig. 6D).

**Figure 7:**
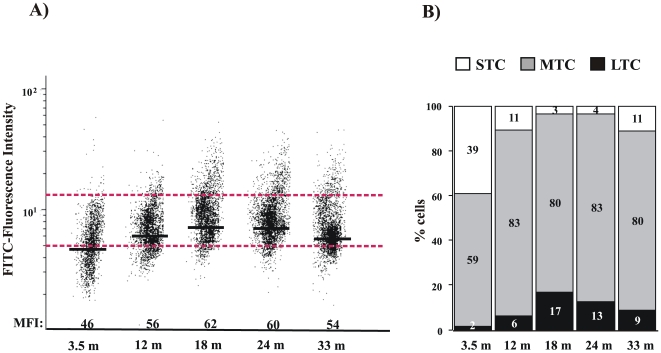
Dynamic of telomere length in zebrafish caudal fin assayed by Flow-FISH. **A**, Representation of the zebrafish caudal fin cells distribution (clip 1) according to their telomere length., Medium Fluorescence Intensity (MFI) is indicated for each age, (n = 5). The same trend was observed in the three independent experiments. **B**, Graphic representation of the percentage of cells with long telomere (LTC), medium telomere (MTC), and short telomere (STC), delimited by dotted red lines, from clip1 at different ages.

The incomplete and deficient fin regeneration in old zebrafish might be correlated with the decreased telomerase expression and telomere length observed at this stage of life. In order to establish whether the upregulation of telomerase expression during the regeneration process has any physiological consequence, we measured telomere length in caudal fin tissues at different fish ages before and after the regeneration process. We found using flow cytometry-fluorescence in situ hybridization (Flow-FISH: Fig. 7A–B) that the telomere length of cells from the caudal fin increased during development until a critical age of 18 months, when it gradually decreased. While shortest telomeres were elongated during the life cycle, the percentage of longest telomeres increased until 18 months (Fig. 7B). These results are in agreement with our TRF and Q-FISH data using whole organisms in three zebrafish genotypes (Figs. 4 and 5).

**Figure 8:**
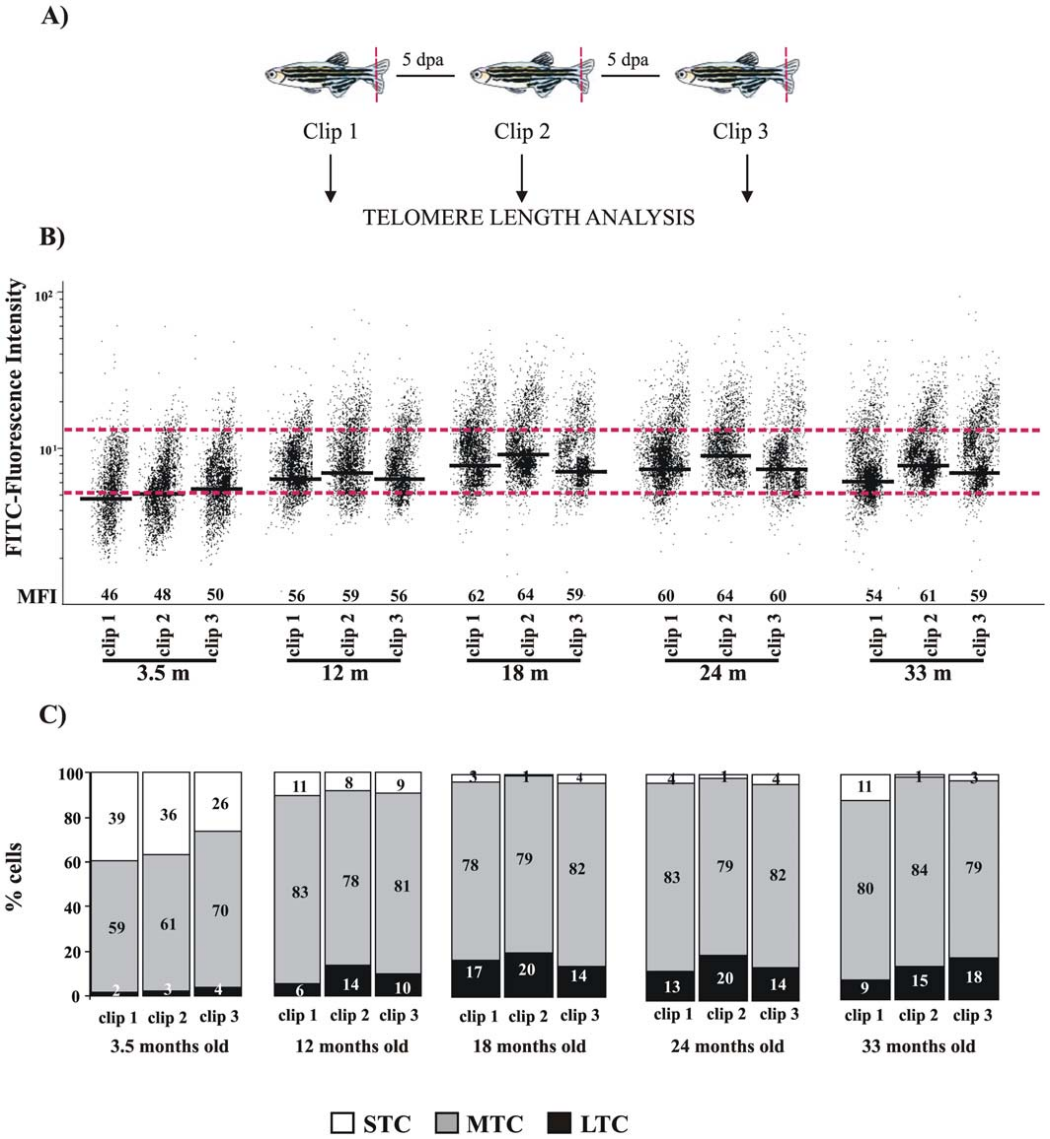
Behaviour of telomere length during fin regeneration by Flow-FISH assay. **A**, Experimental design of the Flow-FISH assay. Clip 1 (1^st^ fin excision), clip 2 (2^nd^ excision) and clip 3 (3^rd^ excision). **B**, Representation of the zebrafish caudal fin cells distribution (clip 1, clip2 and clip3) according to their telomere length. MFI is indicated for each age, (n = 5). The same trend was observed in the three independent experiments **C**, Graphic representation of the percentage of cells with long telomere (LTC), medium telomere (MTC), and short telomere (STC), delimited by dotted red lines, from clip1, clip 2 and clip 3, at different ages.

We next sought to determine the effects of several amputations in telomere behaviour. When the caudal fin was amputated (clip one) and allowed to regenerate for 5 days (clip two, Fig. 8), we found that the average telomere length increased at all ages, as indicated in Figure 8B by the mean fluorescent intensity (MFI). Strikingly, while, very young fish (3.5 months old) were able to further increase their telomere length after a third amputation, older fish showed weak telomere attrition but were able to maintain their original telomere length. The inefficient activation of telomerase expression in oldest fish might be responsible for this behaviour. We also observed that short telomeres decreased, whereas long telomeres increased, after the second clip. In the third clip, however, the percentage of cells with short telomeres increased again in older fish. Collectively, these results suggested that the upregulation of telomerase expression was sufficient to increase telomere length during the rapid cellular proliferation associated with the regeneration process, but after reclipping telomere length was not maintained, except in very young fish (3 months old) that show a robust telomerase induction.

## Discussion

Although, it has been extensively shown that telomeres and telomerase are involved in mammalian aging and cancer promotion and that the zebrafish is now widely used as a good model organism for assessing these complex processes [3], [4], [49], so far there have only been a few incomplete studies about telomeres and telomerase biology in this species. The zebrafish, with a life-span of 3 years, is the only vertebrate system in which telomerase function can be studied in a high-throughput manner. We used this species to perform an exhaustive study of telomerase expression and telomere length among a broad range of tissues throughout its life.

A high level of *TERT* gene expression has been reported in almost all zebrafish tissues in contrast to what happens in the corresponding mammalian tissues [50]. Our results show that telomerase is expressed in all tissues tested at different stages of life (larvae, juvenile, adult and old fish). However, the expression of TERT mRNA drastically decreased in all tissue examined of old fish (more than two years old), with the exception of the muscle. These results are in line with published data in which continuously proliferating myocytes have also been observed although there was no lipofucsine granule accumulation in the muscle of zebrafish with advancing age [51]. These data correlated well with the presence of telomerase activity in all tissues examined of adult fish of different ages and with the significant decrease of telomerase activity in old zebrafish samples, with the exception of muscle and ovary samples.

Despite the observed differences in telomerase expression between mammalian and zebrafish, we detected common transcriptional factor binding sites in both promoters. We observed several Sp1 binding sites, *hTERT* has at least nine Sp1 binding sites dispersed along the 4 kb promoter, whereas *zfTERT* has 16 Sp1 binding sites. The clustering of Sp1 binding sites is a common event in promoters of TATA-less genes [40], [52] such as *hTERT* and *zfTERT*. Another common feature of both promoters is the presence of c-Myc binding sites. Although *zfTERT* has only one site located at 1.200 upstream of the start site and *hTERT* has several for this transcriptional factor, the induction of *zfTERT* promoter in zebrafish embryos by c-Myc is similar to that found with *hTERT* [40]. Similarly, the NF-κB transcription factor also induces the *zfTERT* promoter, suggesting that the zebrafish is also a good model for studying the influence of inflammation on TERT expression and cancer development.

In all the analyzed strains, we observed an increased telomere length from larvae to adult fish and a significant telomere shortening in aged fish. Our results are not in agreement with recently published data [44] in which, using a TRF assay, the authors conclude that telomeres do not shorten with age. These discrepancies might be explained by the methodology used. It is well-known that the TRF technique is not sensitive enough for detecting changes in telomere length and it is difficult to detect short telomeres, something that is crucial in these aging studies [53]. Therefore, we combined this technique with the most accurate Q-FISH technique. Moreover, all our studies have been performed using whole organisms from various strains obtained from different stock centres in order to avoid a particular telomere length associated with a strain and/or to a given stock centre. These results were also confirmed using a specific tissue, such as the caudal fin, where we observed by Flow-FISH that telomere length increased until 18 months old and gradually decreased after this age. Overall, our results showed that the three wild type strains analyzed showed slightly different telomere lengths. TL is the genetic background showing the shortest telomeres and such differences may have an impact on cancer susceptibility and aging. In fact, we have observed that the life-span of the TL background is less than 3.5 years, while the AB and WIK strains have longer life-spans in our zebrafish facility. Further studies should be performed to establish whether these differences are important in aging and cancer processes.

In this study, we have also established that there is a direct relation between the levels of telomerase expression, telomerase activity and telomere length in zebrafish. Haploinsufficiency for TERT leads to premature telomere shortening in human and causes the aging disease known as dyskeratosis congenita [54]–[55]. This means that telomerase levels control telomere length in human and in zebrafish, and therefore, high TERT expression might prevent telomere erosion and delay senescence in adult animals.

In contrast to mammals, lower vertebrates have a remarkable capacity to regenerate complex structures after damage, including heart, spinal cord, retina and fins. This process involves progenitor cells/resident stem cells [56], [57]. In mammals, telomerase is expressed in germ cells and in the stem cell compartment of several adult tissues. It has been proposed that telomerase may be important for tissue regeneration after injury. In fact, phenotypes associated with premature loss of tissue regeneration, including the skin (hair loss, hair greying, decreased wound healing) are found in mice deficient for telomerase [58]–[60]. Our results showed that the zebrafish was able to regenerate the amputated fin at all ages but the fish with the lowest level of telomerase expression; i.e. older fish, had severely impaired fin regeneration. Indeed, the efficiency of regeneration showed a direct correspondence with telomerase expression (3days/young fish versus 12days/old fish). In fact, we have observed an increase in TERT expression in caudal fin, when amputated in young (3 month old) and old fish (24 month old), but only significant in young fish (58% of upregulation). Therefore, young fish responded better than old fish after injury. Importantly, the upregulation of telomerase expression was correlated with telomere length behaviour after reclipping, as the different age groups responded to the injury lengthening their telomeres. Our data were consistent with the idea that telomeres would need to be maintained during increased cell proliferation associated with tissue renewal. Although the strong upregulation of telomerase expression observed in 3 month old fish did not correlate with a strong telomere length increase, this group was the only one able to elongate their telomeres after consecutive amputations. However, fish older than 3 months, although showing an increase in telomere length after a second clip, are not able to maintain this elongation after a prolonged injury (clip 3). This behaviour might be related to the inefficient activation of telomerase expression in fish older than 3 months. Curiously, whereas the oldest fish had a similar telomeric response to adult fish, they showed incomplete and deficient fin regeneration. However, regeneration is a complex process in which many genes/factor are involved. Therefore, further studies are required to clarify the role of telomerase and telomere length in regeneration. Further gain- and loss-of-function experiments for two telomerase components will shed light on the role of telomerase in regeneration.

**Figure 9:**
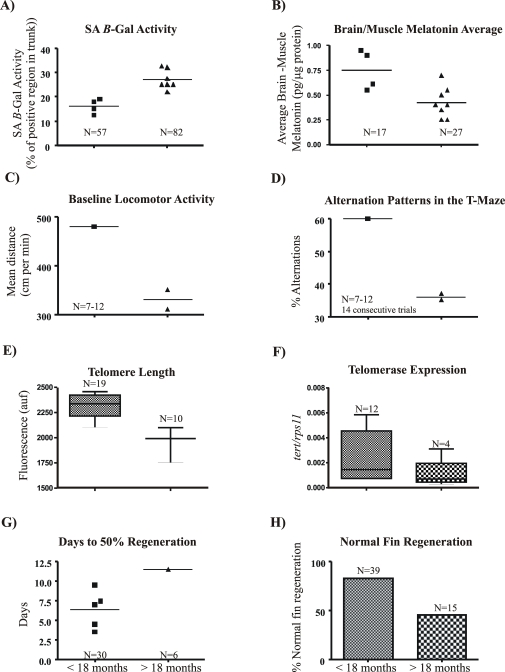
Potential aging biomarkers in the zebrafish. All the biomarkers shown are able to discriminate two age groups. The data were collected from different studies cited in our manuscript. We show the average for each aging marker. The data was divided in two groups:. fish younger than 18 months are all depicted with squares and fish older than 18 months with a triangle. The total fish number used for these two groups was indicated. **A**, β-Gal activity marker [61] (Kishi et al. 2008). **B**, Brain/Muscle Melatonin [63] (Tsai et al. 2007, average of melatonin levels in muscle and brain of different age fish groups). **C–D**, Changes in cognitive function [62], **C**, Baseline locomotors activity level, **D**, Alternation pattern for the choice of the short arms in the T-maze. **E**, Telomere length (our data). **F**, Telomere expression (this manuscript). **G–H**, Regeneration experiments [63 and this manuscript].

Another important insight of our study is the declined telomerase expression, telomere length and regeneration capacity as biomarkers for aging in zebrafish. Until now, SA B-Gal activity, melatonin deficiency and cognitive function were all used as aging biomarkers [61]–[63]. All these biomarkers, together with the ones described in this study, can discriminate between two age groups; that is, those younger than 18 months old and older than 18 months old (Figure 9). These results indicate that telomere length, telomerase expression and regeneration capacity are highly dependent on zebrafish age and, therefore, they are useful for evaluating the aging process of zebrafish.

Further studies are necessary to establish if all the cells express telomerase or only a specific population. A transgenic zebrafish expressing a reporter driven by *TERT* promoter might be useful for identifying *in vivo* cells with high/low telomerase activity, i.e. progenitor- stem cells/aging cells. Although there are obvious differences between human and zebrafish, such as the high expression of *TERT* alongside its life-span, both species show declined telomere expression and telomere length with aging. Their promoters are up-regulated with the same intensity and by similar key transcription factors. We therefore propose that the zebrafish can be used to identify genes and drugs that affect the ability to restore aging phenotypes using telomere length or telomerase expression, which have been identified as good aging biomarkers in this study.

## Materials and Methods

### Ethics Statement

The experiments performed comply with the Guidelines of the European Union Council (86/609/EU) and the Bioethical Committee of the University Hospital “Virgen de la Arrixaca” (Spain) for the use of laboratory animals.

### Maintenance of zebrafish

Zebrafish (*Danio rerio*), AB (from the Zebrafish International Resource Centre (ZIRC), WIK (from ZIRC and Tubingen Zebrafish Stock Centre), TE and TL (Tubingen Zebrafish Stock Centre), were maintained in recirculating tanks following instructions from “The zebrafish handbook: a laboratory use of zebrafish, *Brachydanio rerio*”. Adult fish were maintained at 26°C, with a light:dark cycle of 14∶10 hours and were fed twice daily, once with dry flake food (PRODAC) and once with live arthemia (MC 450, IVE AQUACULTURE). Zebrafish embryos were maintained in egg water at 28.5°C and were fed at 5days with NOVO TOM and with live arthemia at 11 days of life.

The experiments performed comply with the Guidelines of the European Union Council (86/609/EU) and they were approved by the Bioethical Committee of the University Hospital Virgen de la Arrixaca (Spain) under approval number (PI06/FIS0369/040706).

### The Analysis of Zebrafish Telomerase Expression

Total RNA was extracted from whole zebrafish embryos, whole zebrafish adults at several ages and different zebrafish tissues at several ages with TRIzol Reagent (Invitrogen), following the manufacturer's instructions and treated with DNase I, Amplification grade (1 unit/µg RNA, Invitrogen). The SuperScript III RNase H− ReverseTranscriptase (Invitrogen) was used to synthesize first strand cDNA with oligo-dT18 primer from 1 µg of total RNA at 50°C for 60 min.

Real-time PCR was performed with an ABI PRISM 7700 instrument (Applied Biosystems) using SYBR Green PCR Core Reagents (Applied Biosystems). Reaction mixtures were incubated for 10 min at 95°C, followed by 40 cycles of 15 s at 95°C, 1 min at 60°C, and finally 15 s at 95°C, 1 min 60°C, and 15 s at 95°C. For each mRNA, gene expression was corrected by the rRNA11S content in each sample. The primers used were ZfTERT F2: 5′-CGGTATGACGGCCYATCACT-3′ and ZfTERT R1:5′-TAAACGGCCTCCACAGAGTT-3′ for zebrafish TERT, and F:5′-ACAGAAATGCCCCTTCACTG-3′ and R:5′-GCCTCTTCTCAAGGTTG-3′ for 11S rRNA.

### Cell isolation

Zebrafish of different genotypes and stages were anesthetized with 0.05% benzocaine, briefly rinsed in 0.5% chilled bleach, crushed and incubated in PBS supplemented with antibiotics for 30 min., centrifuged (600 g, 5 min.), incubated in Tripsine (0.5 mg/ml)/EDTA (0.1 mg/ml) in PBS for 1 min., centrifuged (600 g, 5 min.) and then incubated in Colagenase (0.5 mg/ml) in RPMI medium supplemented with CaCl_2_ 2H_2_0 (0.7 mg/ml) for 30 min. The cell suspensions were obtained by pipetting, smashing and finally filtering the digested tissues through a 100 µm mesh, washed and resuspended in PBS.

### TRF analysis

Telomere length assay was modified from Blasco *et al*., 1997 [43]. Cells were isolated and embedded in agarose plugs following instructions from the supplier (CHEF agarose plung kit from Bio Rad). Cells or DNA embedded in agarose plugs was digested with *Mbo*I and electrophoresed through 0.5% agarose gels in 0.5X TBE. Separation was for 6 hr at 120 V. The gel was blotted and probed with a 1.6 kb fragment containing the sequence (TTAGGG)n. Southern blot was hybridized to radioactively labelled probe at 65°C in 0.01 g ml^−1^ BSA, 200 mM sodium phosphate, 15% formamide, 1 mM EDTA, 7% sodium dodecyl sulphate (SDS). Filter (Hybond N+ Amersham Pharmacia Biotech) was washed three times in 0.2 X SSC, 0.1% sodium dodecyl sulphate (SDS) at 65°C for 30 min.

The telomeric specific probe was labelled with [α-32P]-dCTP using Ready-to-Go DNA labelling beads according to the supplier's instructions (GE Healthcare).

### Interphase Q-FISH

Q-FISH on interphases was performed as described in Canela *et al*., 2007 [48]. Cell suspensions were obtained as described before. Cy3 and DAPI images were captured at 100x magnification using a Nikon Digital Camera DXM 1200C on a Nikon Direct Eclipse fluorescence microscope. Telomere fluorescence signals were quantified using the TFL-TELO program (from Peter Lansdorp, Vancouver, Canada).

Telomere fluorescence values were converted into kb by external calibration with the HeLa L cell line with known telomere length of 23.82 kb and with the L5178Y-S, L5178Y-R lymphocyte cell lines with known telomere lengths of 10.2 and 79.7 kb, respectively.

### Flow-FISH

One million of cells from each fin sample obtained as described before were washed in 2 ml PBS supplemented with 0.1% BSA. Each sample was divided in two replicate tubes: one pellet was resuspended in 500 µl hybridization buffer and another in hybridization buffer without FITC-labeled telomeric PNA probe as negative control. Samples were then denatured for 10 minutes at 80°C under continuous shaking and hybridized for 2 h in the dark at room temperature. After that, the cells were washed twice in a washing solution (70% deionized Formamide, 10 mM Tris, 0.1% BSA and 0.1% Tween-20 in dH_2_O, pH 7.2). The cells were then centrifuged, resuspended in 500 µl of propidium iodide solution, incubated 2 h at room temperature, stored at 4°C and analyzed by flow cytometry within the following 48 h.

### Telomerase Activity Assays

The TRAPeze® XL Telomerase Detection Kit (Millipore, Cat. #S7707) was used to qualitatively measure the telomerase activity of whole zebrafish embryos extracts. The protein extracts were obtained according to the manufacturer's instructions. Human carcinoma cells (included in the telomerase detection kit) were used as a positive control. As a specific negative control, the higher protein concentration assayed of every sample extract was incubated with 1 µg of RNAse A (QIAGEN) at 37°C for 20 min.

To quantitatively determine the telomerase activity of both whole zebrafish larvae and organ extracts from four fishes per group of 6, 12 and 30 months old, a real-time quantitative TRAP (Q-TRAP) analysis was performed as described by *Herbert et al* (31). The protein extracts were obtained as described by the authors. For making the standard curve, a 1∶10 dilution series of telomerase-positive sample (human carcinoma cells) was used. To quantify the telomerase activity, PCR amplification was performed as indicated by the authors. After PCR, real-time data were collected and converted into Relative Telomerase Activity units performing the calculation: RTA of sample  = 10(Ct sample-*Y*int)/slope. The standard curve obtained was: y = −3.2295x+23.802.

### Tail Regeneration Assay

Zebrafish from AB strain at various stages (1.5, 3, 6, 18 and 24 months old) were anesthetized with 0.05% benzocaine. The fin tissue was removed within approximately 2 mm of the base of the caudal peduncle using a razor blade. The fish were allowed to recover and the animals were returned to recirculating water heated to 30°C for the duration of the experiment. Each fish was tracked individually to calculate regeneration progress over time. Zebrafish fins were imaged before amputation and again on day 3, 5, 8 and 14 postamputation. Percent fin regeneration was determined based on the area of regrowth divided by the original fin area ± standard error.

To study the telomere length in caudal fin regeneration by flow-FISH assay, we obtained, by excision, caudal fin tissue (fin clip 1) from AB strain zebrafish at various life stages (3.5, 12, 18, 24 and 33 months old) as described previously. A second and third excision of fin tissues were repeated with 5 days intervals to obtain regenerated tissues (fin clip2 and fin clip 3, respectively).

### Bioinformatic analysis

The search for the putative transcription factor binding site in the zebrafish telomerase promoter was carried out using TESS (Transcription Element Search System): http://www.cbil.upenn.edu/cgi-bin/tess/tess. The information about the putative transcription factor binding site in the human telomerase promoter was previously described by [52].

### Expression constructs

The zebrafish telomerase promoter fused with the reported fusion EGFP-firefly luciferase (zfpTERT-EGFPLuc) was obtained as described in [42]. In the same way, the human telomerase promoter fused with the reported fusion EGFP-firefly luciferase (hpTERT-EGFPLuc) was obtained. The *Renilla* luciferase expression vector pRL-CMV was purchased from Promega. The NF-κB:luciferase reporter and the c-Myc expression construct were kindly provided by Dr. R. Hay and Dr. S. Gonzalez respectively.

### Microinjection

Firefly and *Renilla* reporter plasmids (100 and 10 pg/egg, respectively) alone or with the cMyc expression construct (100 pg/egg) or with the phenol-extracted genomic DNA from *Vibrio anguillarum* ATCC19264 cells (*Va*DNA) as a PAMP (1 ng/egg) were mixed in microinjection buffer (0.5x Tango buffer and 0.05% phenol red solution) and microinjected (0.5–1 nl) into the yolk sac of one- to eight-cell-stage embryos using a microinjector Narishige IM-300 [42].

### Luciferase assay

Twenty-four hours post-microinjection, the firefly and *Renilla* luciferase activities were determined using the Dual-Luciferase Reporter Assay kit (Promega) as described in *Alcaraz-Pérez et al. 2008*. To discriminate the activity of the two types of luciferases, an Optocomp I Luminometer (MGM Instruments) was used.

### Statistical analysis

Data were analyzed by ANOVA and a Tukey multiple range test to determine differences between groups. The differences between two samples were analyzed by Student's *t* test.
